# Retrospective analysis of mortality and quality of life after hip disarticulation or hemipelvectomy: a report on 15 patients

**DOI:** 10.1007/s00402-023-04783-4

**Published:** 2023-02-01

**Authors:** Melanie Schindler, Susanne Baertl, Nike Walter, Siegmund Lang, Dominik Szymski, Volker Alt, Markus Rupp

**Affiliations:** Department of Trauma Surgery, University Hospital Regensburg, Franz-Josef-Strauss-Allee 11, 93053 Regensburg, Germany

**Keywords:** Hip disarticulation, Hemipelvectomy, Amputation, Infection, Tumor, Survival

## Abstract

**Background:**

Hip disarticulation and hemipelvectomy are defined as major ablative amputations of the lower limb. Due to the small number of patients, little is known about the outcome and follow-up.

**Aims:**

We aimed to assess (1) reasons for performed major ablative surgeries such as hip disarticulation and hemipelvectomy in a German center for trauma and orthopedic surgery. (2) In addition, mortality and quality of life after hip disarticulation and hemipelvectomy as well as (3) patient and treatment characteristics should be investigated.

**Methods:**

During a period of twelve years, 15 patients underwent hip disarticulation or hemipelvectomy. Mortality, EQ-5D-3L quality of life by EQ-5D-3L and time-trade-off (TTO), VAS, cause of disarticulation, length of hospital stays, revisions, comorbidities, Charlson comorbidity index (CCI), and ASA score were evaluated retrospective for all patients.

**Results:**

The overall mortality rates were 26.7% at 30 days, 60.0% after one year and 66.7% after three years. The five surviving patients reported about moderate problems in the EQ-5D-3L. The average VAS score reached 45 (range 15–65). The mean TTO was 9.8 (range 6–12). Indications for amputation were infection (*n* = 7), tumor (*n* = 6), trauma (*n* = 1) and ischemia (*n* = 1).

**Conclusion:**

Hip disarticulation and hemipelvectomy are followed by a high postoperative mortality. Quality of life of the affected patients is impaired in long-term follow-up. Especially amputations performed due to infections show high mortality within one month after surgery despite average young age and low CCI. Surgeons should be aware of this devastating outcome and extraordinary vigilant for these vulnerable patient cohorts.

## Introduction

Hip disarticulation and hemipelvectomy are defined as major proximal lower limb amputations. The most common indications for these mutilating surgeries include periprosthetic joint infection following hip arthroplasty, necrotizing fasciitis (NF), malignant musculoskeletal tumors, severe trauma and limb ischemia due to peripheral arterial occlusive disease (PAOD) [1]. Fortunately, hip disarticulation and hemipelvectomy need to be performed only rarely. In the USA, from 1988 to 1996, hip disarticulations accounted for 0.4% of lower extremity amputations and pelvic disarticulations for 0.1% [1]. Recently a decrease in the incidence for major amputations, which are defined as amputations above the transmalleolar level, has been observed. Intriguingly, a notable rise of major amputations close to the pelvis (hip disarticulation + 39.7%, hemipelvectomy + 122.5%) has been elucidated, in the adult German population [2].

Although both surgeries are of incredible importance for the further life of the patients and are most often performed as the last therapy option to preserve the patient's life, little is known about the outcome and follow-up of the patients concerned.

There are only a few studies that evaluated mortality after major proximal lower limb amputation, and the results remain controversial. Unruh et al. [3] reported an overall mortality rate of 44.0% over eleven years in their series of 34 patients. In contrast, Tanner et al. [4] found a lower hip disarticulation mortality with 16.7% at 30 days, 29.2% at 6 months and 31.3% at one year. For patients suffering bone sarcoma who required hemipelvectomy, overall survival has been described as 91 months [5].

Therefore, the present study aimed to broaden knowledge about this special and small patient group. Therefore, we aimed to assess (1) reasons for performed major ablative surgeries such as hip disarticulation and hemipelvectomy in a German center for trauma and orthopedic surgery. (2) In addition, mortality and quality of life after hip disarticulation and hemipelvectomy as well as (3) patient and treatment characteristics should be investigated.

## Materials and methods

This study is a single-center retrospective analysis of patients who underwent either hip disarticulation or hemipelvectomy between January 2010 and September 2022 at a German center for trauma and orthopedic surgery. Patients were identified using the Operation and Procedure Classification System (OPS) code 5-864.0, 5-864.1, or 5-864.2. All patients were retrospectively characterized and evaluated using clinical records for sex, age at surgery, cause of disarticulation, length of hospital stay, revisions, mortality, comorbidities, Charlson comorbidity index (CCI) and the American Society of Anesthesiologists (ASA) score.

“Health-related quality of life” (QOL) was assessed with the five-dimensional EuroQol score (EQ-5D) [6] in the form of telephone self-report at the last follow-up. The EQ-5D questionnaire includes five dimensions of health status: mobility, self-care, usual activities, pain/discomfort, and anxiety/depression. The response options for each of the five dimensions are: no problems, moderate problems and extreme problems, resulting in 243 possible quality-of-life conditions. The EQ-5D questionnaire also includes a visual analog scale (VAS) that represents respondents’ self-rated health status on a scale (0–100), with higher scores indicating higher health-related quality of life. The standardized Time Trade-off (TTO) key score with a scale from 1 (best) to 3 (worst) was used to assess health-related quality of life. Informed consent was taken from all patients and participation was voluntary.

IBM SPSS Statistics 25 (SPSS Inc., Chicago, IL, USA) was used for the analysis. Demographic and clinical characteristics were expressed as mean and standard deviation. Survival and influencing factors were calculated using the Kaplan–Meier analysis (log-rank (Mantel-Cox)). The statistical significance of the association between each parameter and recurrence-free survival was determined using the ANOVA test. *T* tests were used to compare the means in the groups. *P* value < 0.05 was considered significant. The study was approved by the local Ethics Committee (file number 20-1680_2-101).

## Results

**Fig. 1:**
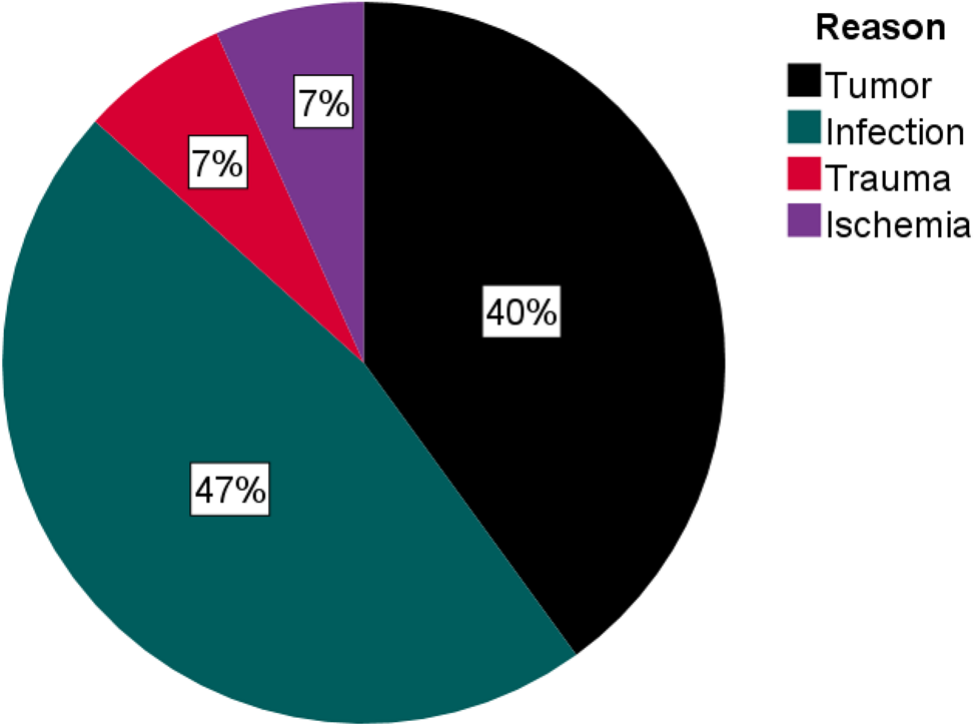
Reason for amputation

In total 15 patients could be identified and included into the study. Eight women (53.3%) and seven men (46.7%) with a mean age of 60.5 ± 16.3 years at surgery (range 21–82 years) were included. Proximal major limb amputations were performed electively in eight patients. Nine different senior surgeons performed the operation. The remaining patients (*n* = 7) required emergency surgery. The indications were infection (*n* = 7), tumor (*n* = 6), trauma (*n* = 1) and ischemia (*n* = 1) (Fig. 1; Table 1).

**Table 1:** Patient characteristics

Variable	Tumor *n* = 6 (40%)	Infection *n* = 7 (46%)	Trauma *n* = 1 (7%)	Ischemia *n* = 1 (7%)
Age in years Mean (range)	69 (49–82)	50 (21–66)	71	72
Sex (male/female)	5/1	2/5	0/1	0/1
Operation (Hemipelvectomy/Hip disarticulation)	3/3	1/6	0/1	0/1
Operation time in minutes Mean (range)	185 (102–359)	150 (82–508)	90	112
Hospital stay in days Mean (range)	35 (7–76)	78 (3–184)	13	43
Post-surgery survival in days Mean (range)	645 (129–1400)	865.6 (1–3103)	217	13
Mortality (percentage)	4 (67%)	4 (57%)	1 (100%)	1 (100%)
CCI Score Mean (range)	4 (2–6)	2 (0–7)	3	1
ASA Score Mean (range)	3 (2–6)	3 (2–5)	3	3
VAS Mean (range)	30 (15–45)	55 (50–65)	–	–

**Fig. 2:**
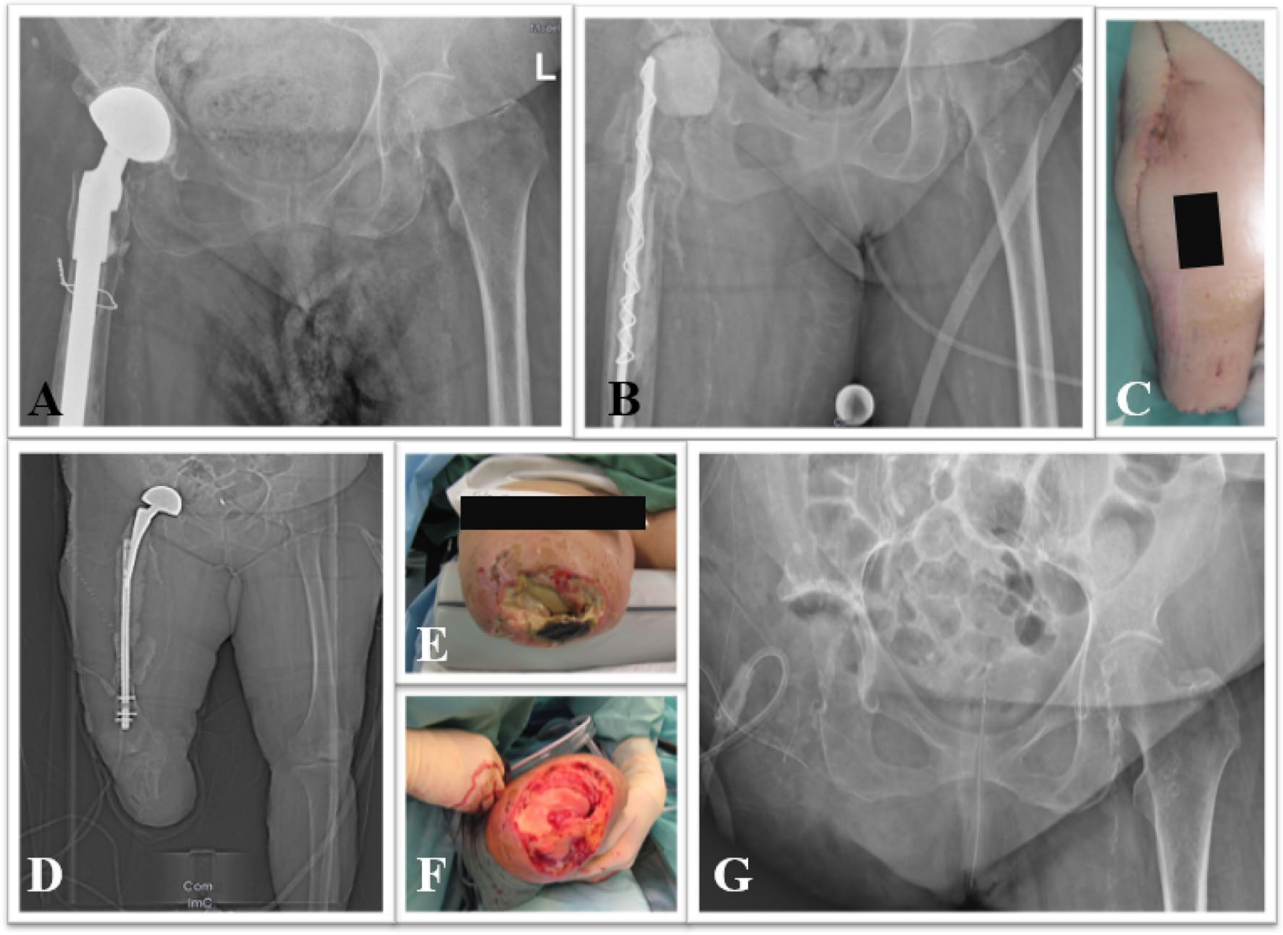
66 year-old female patient with FRI after open reduction and internal plate fixation distal fibula fracture, end-stage peripheral artery disease and consecutive concomitant PJI of the hip. **A** X-ray of the pelvis with revision endoprosthesis prior to explantation (a.p. view). Puncture revealed vancomycin-resistant *Enterococcus faecalis*. **B** Postoperative X-ray after implantation of a PMMA spacer. Below knee amputation was indicated due to severe ankle osteomyelitis and lateral soft tissue defect. **C** Image after below knee amputation. **D** CT scout after below knee amputation.** E** Infection of the amputation stump after three months. **F** Intraoperative image after debridement. Due to persistent wound infection and soft tissue defect hip discarticulation was performed.** G** Postoperative X-ray after hip disarticulation

The cases in which infection occurred were NF (*n* = 3), periprosthetic joint infections (PJI) (*n* = 2), stump infection (*n* = 1) and pressure ulcer (*n* = 1). Hip disarticulation (*n* = 2) and external hemipelvectomy (*n* = 1) were performed for NF with the following detecting microorganisms: One patient with a polymicrobial infection caused by *Escherichia coli, Staphylococcus aureus, Proteus vulgaris and Streptococcus hemolyticus* died one day following hemipelvectomy from multiple organ failure. Another patient previously underwent transtibial amputation for NF with bacterial evidence of *Streptococcus pneumonia*. In a third patient *Aspergillus fumigatus* and *Candida glabrata* were evidenced in culture samples. Two hip disarticulations resulted due to PJI of the hip. One microorganism detection of vancomycin-resistant *Enterococcus faecalis *(Fig. 2) and another of *Staphylococcus epidermidis*. After a traumatic transfemoral amputation, a patient suffered a residual limb infection with *Pseudomonas spp*. And hip disarticulation was performed. One hip disarticulation was performed because of soft tissue infection with bacterial evidence of MRSA, 3 MRGN, *Morganella morganii* and *Candida krusei*.

**Fig. 3:**
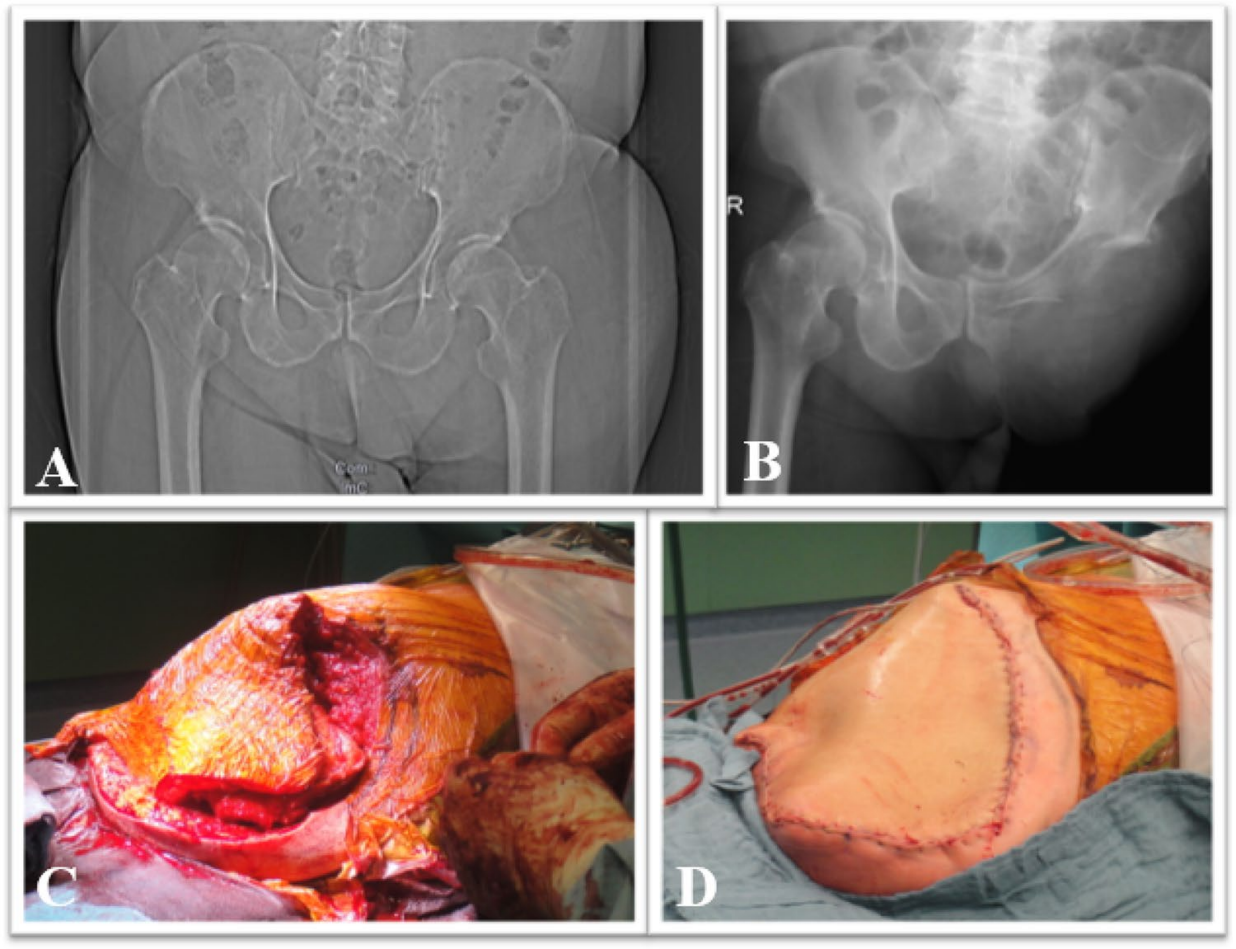
72 year-old male patient with recurrence of unclassified soft tissue sarcoma and performed external hemipelvectomy. **A** Preoperative X-ray of the pelvis (a.p.). **B** Postoperative X-ray of the pelvis(a.p.). **C** Intraoperative image of the external hemipelvectomy before and **D** after wound closure

The most common malignant reason was undifferentiated pleomorphic sarcoma. Of these, two patients underwent hemipelvectomy (one an external and one an internal) and three patients underwent hip disarticulation. One was first amputated above the knee. Finally, an external hemipelvectomy was necessary after recurrence at 23-month follow-up (Fig. 3). The other four patients first underwent hip disarticulation. In one of these patients hemipelvectomy was performed due to tumor recurrence ten months later. Another malignant reason for hemipelvectomy was chondrosarcoma (*n* = 1).

Another reason for hip disarticulation was trauma (*n* = 1). One patient had a femoral neck fracture in an already above knee amputated patient.

The last patient received hip disarticulation after multiple bony amputations (toe than knee disarticulation) and subsequent infection of the amputation stump. PAOD could be identified as reason for the recurrent amputations ending finally in hip disarticulation.

Revision surgeries were performed in five patients (33.3%), mainly due to wound healing disorders (*n* = 3) or local metastasis recurrence at the amputation stump (*n* = 2).

The median postoperative hospital inpatient stay was 54 days and ranged from three to 184 days, whereby the three-day hospital stay resulted from death of the patient. The mortality rate in hospital was 33%. This patient died on average on the 17th postoperative day (range 1–36 d).

### Mortality

**Fig. 4:**
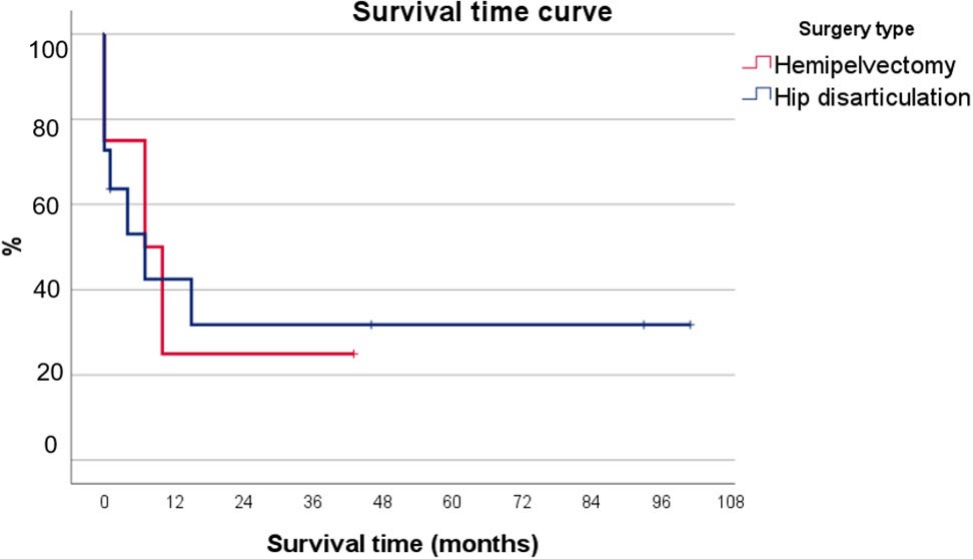
Kaplan–Meier probability plots for survival by surgery type

**Fig. 5:**
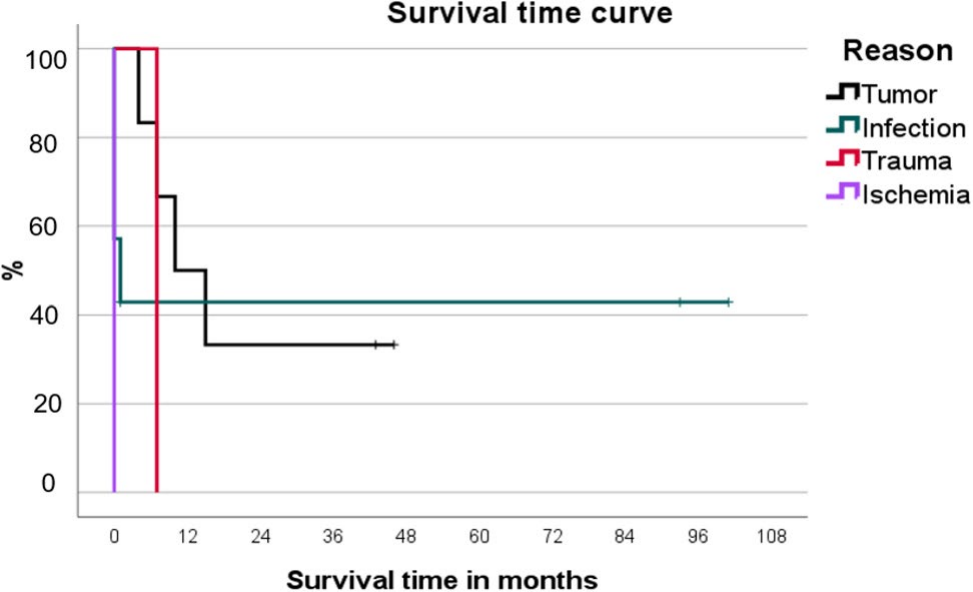
Kaplan–Meier probability plots for survival by indication

Mean overall post-surgical survival time was 22 months (range one day to eight years). The overall mortality rates were 26.7% at 30 days, 60% after one year and 66.7% after three years (Figs. 4 and 5).

### Quality of life

**Fig. 6:**
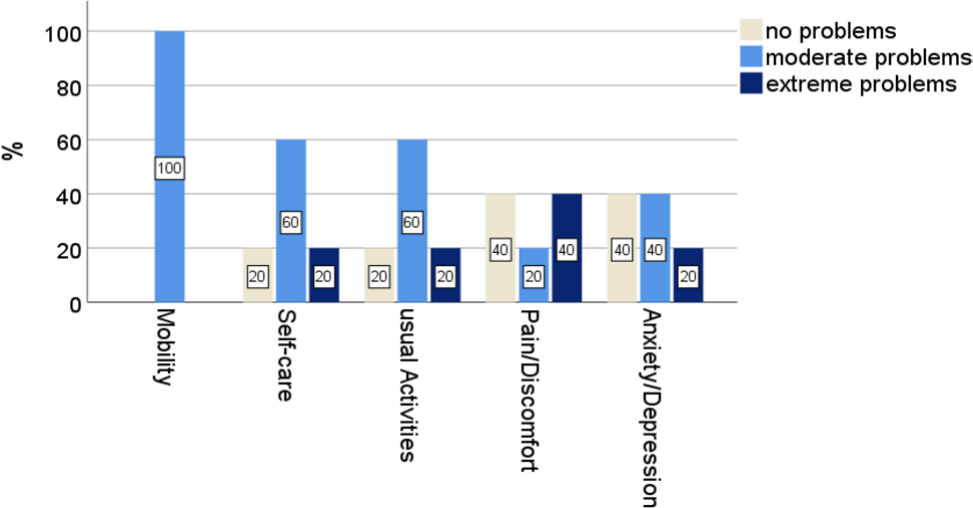
EQ-5D-3L by dimension

The QoL was collected a mean of 57 months after the operation. The mean age was here 56 years and the mean CCI was 1.2. The five surviving patients reported about moderate problems in the life domains: mobility, self-care, usual activities, pain/discomfort and anxiety/depression (Fig. 6). The average VAS score reached 45 (range 15–65). The mean TTO was 9.8 (range 6–12).

## Discussion

The present case series demonstrates that mortality after hip disarticulation or hemipelvectomy is high, regardless of the underlying cause. Furthermore, in the surviving patients a relevant impairment in mobility, self-care, usual activities, pain/discomfort and anxiety/depression could be evidenced. Furthermore, VAS and TTO are useful tools to assess quality of life in patients still alive after hip disarticulation or hemipelvectomy. Both measures demonstrated a relevant reduction in quality of life in the amputated patients.

### Mortality

There are different mortality rates reported in the literature after hip disarticulation or hemipelvectomy. Moura et al. [7] analyzed a series of 16 patients who underwent hip disarticulation over a period of 16 years. Indications included infection (*n* = 6), tumor (*n* = 6), trauma (*n* = 3) and ischemia (*n* = 2). The most frequently detected microorganism in cases of infection was *Staphylococcus aureus* (*n* = 3), followed by *Pseudomonas aeruginosa* (*n* = 2) and *Enterococcus faecium* (*n* = 2). In this case series the most common infections were monomicrobial infection. In our study mainly polymicrobial infections led to amputation. Sarcomas were the dominant type of tumor (chondrosarcoma *n* = 3, pleomorphic sarcoma *n* = 2, basal cell tumor *n* = 1). Moura et al. observed a rate of postoperative complications in seven patients (43.8%) because of superficial infections (*n* = 5), suture dehiscence (*n* = 2) and necrosis of the amputation stump (*n* = 1). Complication rates were significantly higher for emergency procedures than for elective procedures (66.7% versus 38.5%). The complication rate is very similar to our results with 33.3%, while in our analysis the complication rate in relation to emergency and elective surgery is balanced. The overall mortality rates were 31.3% after six months, 43.8% after one year and 50.0% after three years. Overall mortality rates were higher in hip disarticulations due to trauma (66.7%) and tumor (60.0%). The mean overall post-surgical survival time was 200.5 days. Compared to our study, mortality (60% at one year and 66.7% at three years) was higher.

Compared to our results, Unruh et al. [3] reported a higher mortality rate in patients with infections. Twelve of 23 patients with preoperative infection died compared with three of eleven patients without infection. In another study including only emergency surgery for NF, two of five patients died in the postoperative period [8]. Another series of 15 patients published by Zalavras et al. [9] included seven patients with NF and eight patients with persistent infections of the proximal femur. Although one of the seven patients died within a month of the procedure, the authors concluded that this procedure was associated with a “low” mortality rate. This finding is interesting considering that previous reports have reported mortality rates as high as 50% associated with hip disarticulation [3, 10]. In our study, three of seven patients (43%) with infection died within one month after surgery. Of these, two patients had NF and one patient had a pressure ulcer. Compared to the tumor patients, the first patient died four months after operation.

Some studies focused on the mortality of a specific diagnosis, most common on tumor. A retrospective case series collected data from 35 patients who underwent hemipelvectomy between 2000 and 2013. Mean survival time was 32.8 ± 4.6 months and 5-year survival rate was 27%. The survival rate was higher among patients with bone tumors than among those with soft tissue sarcomas [11]. Another study evaluated a mean overall survival time of 70.2 months [12]. The median survival time in our study was 20 months in tumor patients (range 4–46 months, with only 3 of 6 patients getting hip disarticulation.

### QOL

The five survivors in this study reported, on average, moderate problems in mobility, self-care, usual activities, pain- and depression.

Unruh et al. [3] assessed that only four of 19 hip disarticulation survivors in their series used a walker, while twelve used a wheelchair and three were bedridden.

Yari et al. [13] assessed the level of activities, participation and experienced mobility limitations after hip disarticulation and hemipelvectomy. The most common diagnosis for surgery was tumor (78%). The 46 patients (31 hip disarticulation, 15 hemipelvectomy) had relatively high activity and participation levels, but at the same time had limitations in walking, standing up and sitting down, and climbing stairs. Interestingly, the hip disarticulation patients had significantly worse emotional stability than the hemipelvectomy patients.

A study looking at 76 war-related hip disarticulations in Iran determined a VAS of 32.9 ± 33.2. As expected, health-related quality of life in veterans here was significantly lower than in the general population in all subscales except vitality. [14] The average VAS score was 45 (range 15–65), including hemipelvectomy. Also, the mean age of the participants was 19.54 ± 7.96 years, which was different from our study.

### Limitations

The limitations of the present study are its retrospective character and the small and heterogeneous patient group. Although our facility is a level I academic trauma center including a specialized musculoskeletal infection unit as well as sarcoma center, only a limited number of cases could be collected within the last 13 years. However, this study should contribute to the limited number of cases in the literature and widen the knowledge about mortality and reduced quality of life in this vulnerable patient cohort.

## Conclusion

In conclusion, hip disarticulation and hemipelvectomy are major ablative surgeries with high postoperative mortality, especially in patients suffering from infection despite young age and low CCI. Quality of life is impaired in long-term follow-up. Surgeons should be aware of this devastating outcome and extraordinary vigilant for these vulnerable patient cohorts.

## Data Availability

The datasets generated for this study are available on request to the corresponding author.
